# Agriculture along the upper part of the Middle Zarafshan River during the first millennium AD: A multi-site archaeobotanical analysis

**DOI:** 10.1371/journal.pone.0297896

**Published:** 2024-03-28

**Authors:** Basira Mir-Makhamad, Pavel Lurje, Vikentiy Parshuto, Abdurahmon Pulotov, Firuz Aminov, Michael Shenkar, Muminkhon Saidov, Nikita Semenov, Sharof Kurbanov, Sirojiddin Mirzaakhmedov, Khusniddin Rakhmanov, Rita dal Martello, Robert Spengler

**Affiliations:** 1 Department of Archaeology, Max Planck Institute for Geoanthropology, Jena, Thuringia, Germany; 2 Department of Archaeology, Domestication and Anthropogenic Evolution Research Group, Max Planck Institute for Geoanthropology, Jena, Thuringia, Germany; 3 Ancient Oriental Studies Department, Friedrich Schiller University, Jena, Germany; 4 State Hermitage Museum, St Petersburg, Russia; 5 Institute for the Study of the Ancient World, New York University, New York City, New York, United States of America; 6 Institute of History, Archaeology and Ethnography, Academy of Sciences of Tajikistan, Dushanbe, Tajikistan; 7 Institute for the History of Material Culture of the Russian Academy of Sciences, St Petersburg, Russia; 8 Department of Islamic and Middle Eastern Studies, Hebrew University of Jerusalem, Jerusalem, Israel; 9 "New Uzbekistan" University, Tashkent, Uzbekistan; 10 Samarkand Institute of Archaeology, Agency of Cultural Heritage of the Republic of Uzbekistan, Samarkand, Uzbekistan; 11 Department of History and Cultural Heritage, “Silk Road” International University of Tourism and Cultural Heritage, Samarkand, Uzbekistan; 12 Department of Asian and North African Studies, Università Ca’Foscari, Dorsoduro, Venezia, Italy; Washington University in Saint Louis, UNITED STATES

## Abstract

The Zarafshan River runs from the mountains of Tajikistan and terminates in the sands of the Kyzyl-Kum Desert in Uzbekistan; it served as a communication route and homeland for the Sogdians. The Sogdians are historically depicted as merchants existing from the end of the first millennium BC through the first millennium AD. While recent research has provided the first glimpse into cultivation, commerce, communication, and consumption in the Lower Zarafshan, the agricultural heartland of the Middle Zarafshan Basin has remained unstudied. This paper presents the results of archaeobotanical investigations conducted at five ancient urban sites/areas spanning the fifth to the twelfth centuries AD: Kainar (Penjikent citadel), Penjikent (shahristan), Sanjar-Shah, Kuk-Tosh (pre-Mongol Penjikent), and Afrasiab. Collectively, these data show that cereals, legumes, oil/fiber crops, fruits, and nuts were cultivated on the fertile Zarafshan floodplains. In this paper, we discuss evidence for the diversification of the agricultural assemblage over time, including the introduction of new staple crops and fruits into an already complex cultivation system. In addition, we contrast our data with previously published results from sites along the course of the Zarafshan to determine whether there is a dietary difference between pre-and post-Islamic conquest periods at settlements located along the river.

## Introduction

The Zarafshan valley was one of the main agricultural swaths of ancient Central Asia, supporting commercial urban settlements for the past five millennia. The gradient of the valley and the continued summer-long glacial melt provide an ideal situation for irrigated agriculture in an otherwise arid environment. The valley is defined by a heterogeneous geographic landscape with vegetation communities sharply shifting as one rises up the valley slopes, from, fertile upland meadows, foothills, arid deserts, and *Artemisia*-steppe zones. The river originates in the highly glaciated Alai Range of Tajikistan, and today it terminates in the Bukhara Oasis in Uzbekistan. The Zarafshan was a tributary of the Amu-Darya River, which in turn once fed into the Aral Sea, but a reduction in glacial output and heavy water diversion for agriculture have significantly reduced water flow. The Middle and Lower Zarafshan valleys are the main cotton-growing zones of Central Asia today [1]. The valley is composed of a rich floodplain and some of the most arable land in Central Asia, due to its braided river beds and expansive irrigation system. The mean annual precipitation is orographically determined and influenced by the westerlies, ranging between 367 mm (Samarkand) and 432 mm (Penjikent), with greater precipitation accumulation in the spring [2,3]. Penjikent and its surrounding areas, although historically part of the Samarkand region, can be considered part of the Middle Zarafshan piedmont. It is located closer to the mountains, with slightly lower average temperature and slightly higher precipitation, the archaeological complexes possess more elements of highland culture such as handmade vessels; most importantly, the lands above Rabat-i Khoja (medieval Varaghsar) on the border of Tajikistan and Uzbekistan are irrigated by Magian and smaller mountain streams and not by the Zarafshan.

From the second half of the first millennium BC until the eight century AD (twelfth century AD in the Samarkand region), this area was known as Sogdiana. Sogdiana was an agricultural and political center with a “city-state culture” [4], often described by historians as existing at the heart of Central Asian commerce. The region first appears, historically, in the *Avesta* (*Vendīdād*, 1.4; *Yašt* 10.14), Achaemenid inscriptions (DB1.16; XPh 21) dated to the sixth century BC, and was mentioned by Herodotus [5]. Discussing the emergence of professional Sogdian merchants and the origin of their commercial network, Morris suggests that trans-regional exchange was likely restricted to a Kangju ruling class [6]. Historians attest to the expansion of agricultural land from the fifth to the sixth centuries AD in Sogdiana [7]. They also debate over the boundaries of Sogdiana, whether it included the “lands between the middle course of the Amu Darya and the Syr Darya or was limited to the Zarafshan valley” [8]. Almost 80 years ago, Frye [9], critically evaluating textual sources, suggested that Sogdians originally only occupied a small area along the Zarafshan River and Kashkadarya valley, but expanded their range to cover a large area within Central Asia.

**Fig 1:**
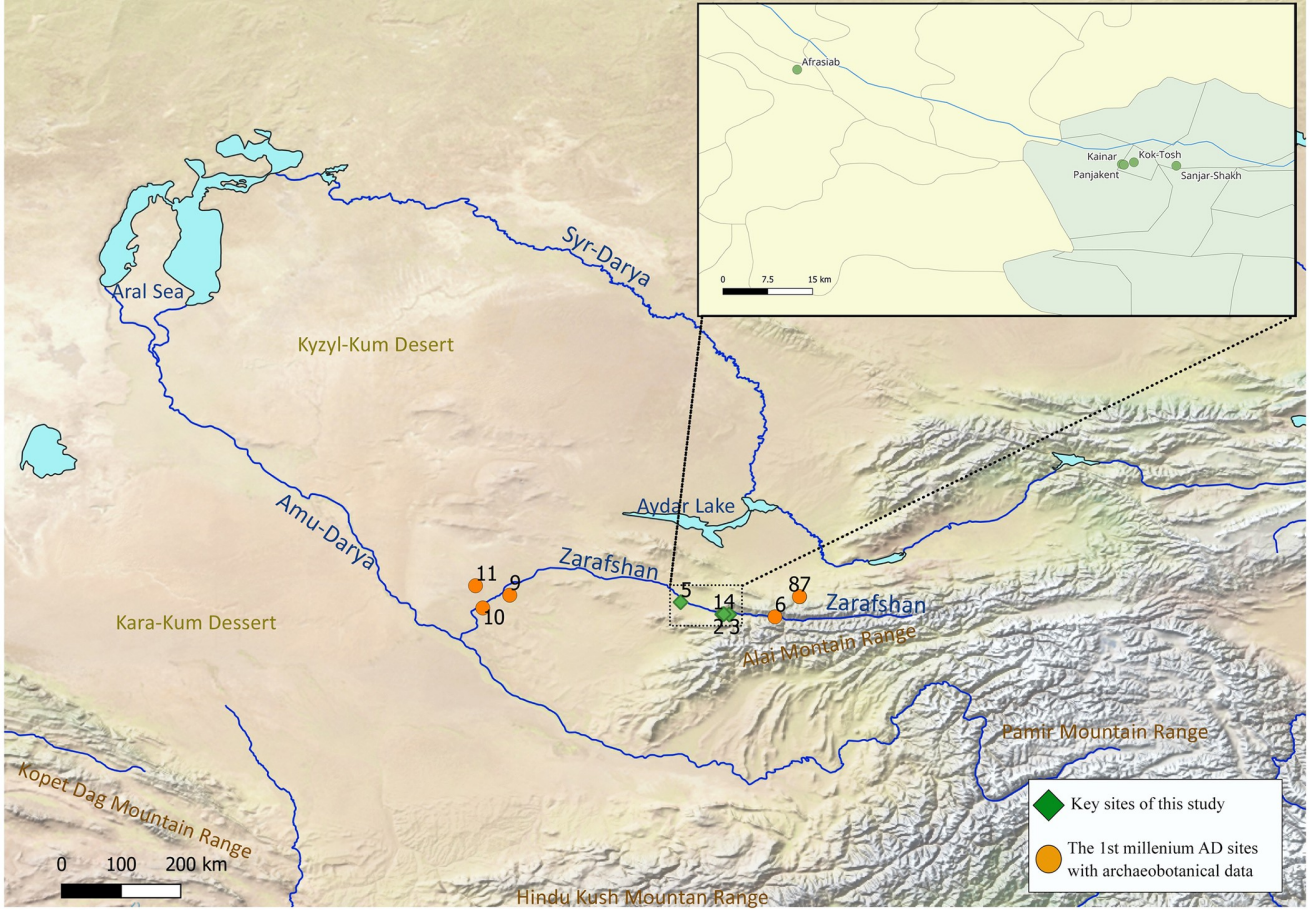
Archaeological sites along the Zarafshan River. 1 –Kainar, 2 –Penjikent, 3 –Sanjar-Shah, 4 –Kuk-Tosh, 5 –Afrasiab, 6 –Mugh, 7 –Urtakurgan, 8 –Chilkhudzhra, 9 –Bukhara, 10 –Paykend, and 11 –Bash-Tepa (Both maps were created in QGIS using Natural Earth Data. Free vector and raster map data @ naturalearthdata.com).

We present the results of archaeobotanical studies at five archaeological sites, situated on alluvial terraces of the Zarafshan River in northwestern Tajikistan and northeastern Uzbekistan: 1) Kainar (the Penjikent citadel), 2) Penjikent (the shahristan), 3) Sanjar-Shah, 4) Kuk-Tosh (pre-Mongol Penjikent), and 5) Afrasiab (Fig 1). We use the site names of Kainar, Penjikent, and Kuk-Tosh to represent distinct archaeological complexes. In this manuscript, we aim to: 1) provide new data about the agriculture of the upper part of the Middle Zarafshan region during the first millennium AD; 2) explore whether there are differences in the Middle and Lower Zarafshan regions; and 3) yield insights into the diet of Sogdians before and after the Islamic conquest. The Islamization of Central Asia, especially from a cultural perspective, was not a singular event, but rather a long process. The first Muslim raids into Transoxiana occurred in the 650s. The main conquest took place between AD 708 and 713 under Qutayba, followed by various struggles, such as the uprising of Dewashtich in AD 722, leading partial withdrawals until pacification under Nasr b. Siyyar in the 740s. The conversion of larger groups of Central Asians to Islam occurred during the Abu Moslem revolt and the establishment of Abbasid rule around AD 750.

### Study area

#### Kainar (5 – 6^th^ centuries AD) and Penjikent (7 – 8^th^ centuries AD)

**Fig 2:**
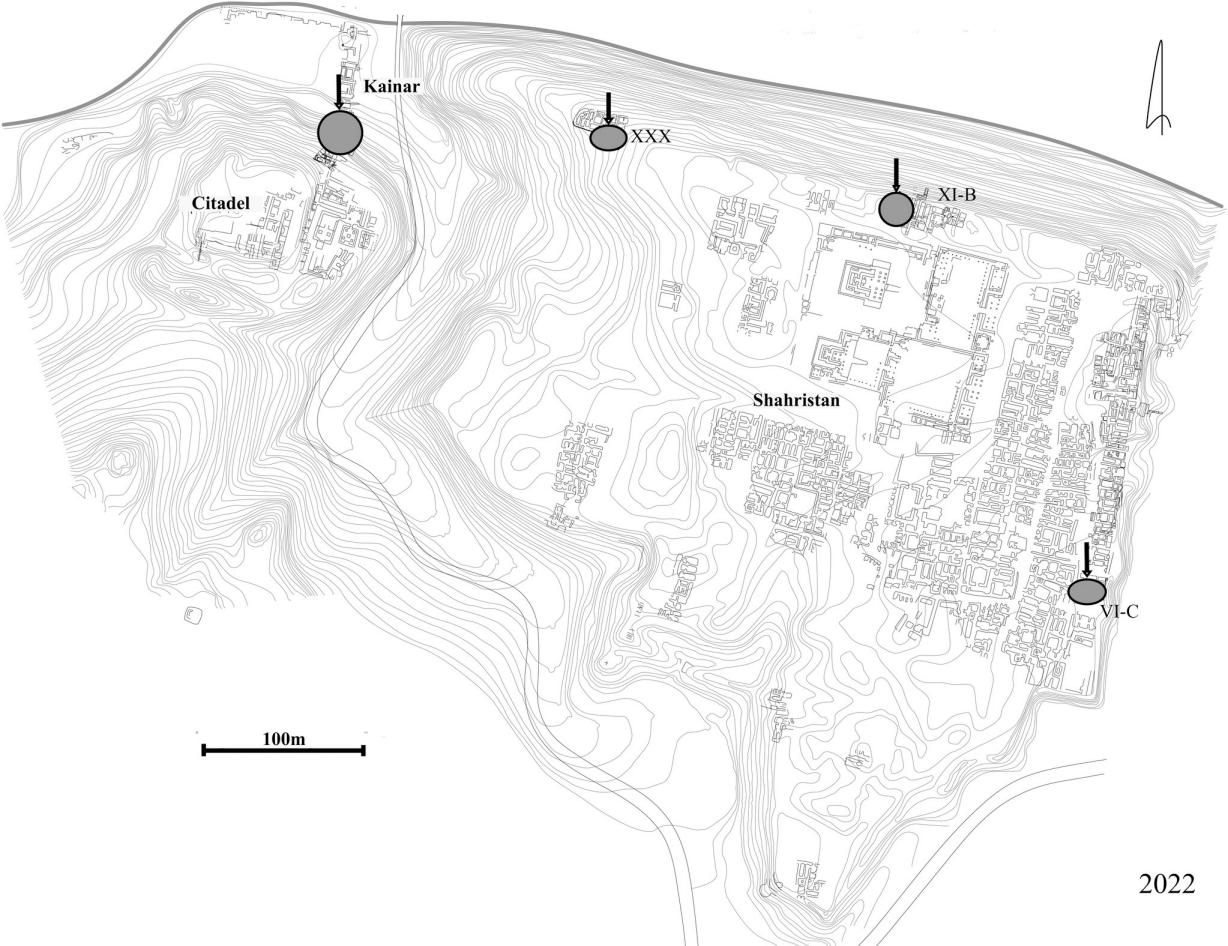
Kainar (citadel) and Penjikent (shahristan) general plan with grey circles where samples were taken.

Ancient Penjikent is sometimes described as the most eastern urban center in a chain of Sogdian great cities, dating from the fifth to eighth centuries AD [10]. Penjikent is located on the southern bank of the Zarafshan River about 60 km to the east of Afrasiab (ancient Samarkand) and about 300 km from Bukhara (Fig 1). It is located on the rim of a high terrace overlooking a fertile valley [10]. The ancient city consists of two distinct parts: a smaller citadel with the area near Kainar spring and the main city or shahristan (Fig 2). The Penjikent citadel to the north of the shakristan is referred to as Kainar (39.488464, 67.616619; 1022 m asl), it lies to the west of the city and was strongly fortified. The citadel of Penjikent was fortified from all sides by city walls, which were regularly rebuilt resulting in repeated encasings of the old walls. Semenov [11] reported archaeological evidence for six construction periods at Kainar, dating from the end of the fourth to the beginning of the eighth centuries AD. We present data here mainly recovered from cultural layers dated to the fifth-sixth centuries AD, both from the northern part of the citadel hill and the Kainar spring area immediately to the north at the bottom of the citadel hill.

The Penjikent shahristan (39.487199, 67.620844; 1051 m asl) was established in the fifth century AD and became one of the prominent cities of Sogdiana. The warm and relatively humid climate, abundant water supply, coupled with fertile loess soil, provided an excellent location for agriculture [3,12]. It was partially burned during the Islamic conquest in September of AD 722, re-inhabited around AD 740 and completely abandoned in the 770s-780s [13]. Before the fire in the AD 722, there was contact with Arabs, the Bedouins were depicted in Dewashtich’s palace, and from Dewashtich’s Arabic letter from Mount Mugh, one can deduce that he, himself, converted to Islam. After 722, the principality of Penjikent ceased to exist, there were no new local coinages, the palace was transformed into the Arab garrison, the city was in ruins and partly burnt. After AD 738, the city of Penjikent was reoccupied, and some of the best-preserved wall paintings come from this period; although, the temples were not in "usual" use. By the 750s the majority of the coins are standard Arab false type; there are no new murals were erected and some of the old ones were deliberately covered or destroyed. The city’s infrastructure did not work properly, and the streets accumulated about 2 meters of garbage during the two decades. Probably, at that time, the city dwellers started to move to the lower terrace, and there are no coins dated to after 770 in the shahristan area of Penjikent. In addition to the Islamic conquest, some scholars believe that shifts in prevailing air masses played a significant role in the abandonment, whereas a shift to a drier climate could have compounded the political unrest [3,12]. However, it is equally likely that people favored unfortified villas to densely populated cities, which lacked water and trees.

#### Sanjar-Shah (8 – 9^th^ centuries AD)

**Fig 3:**
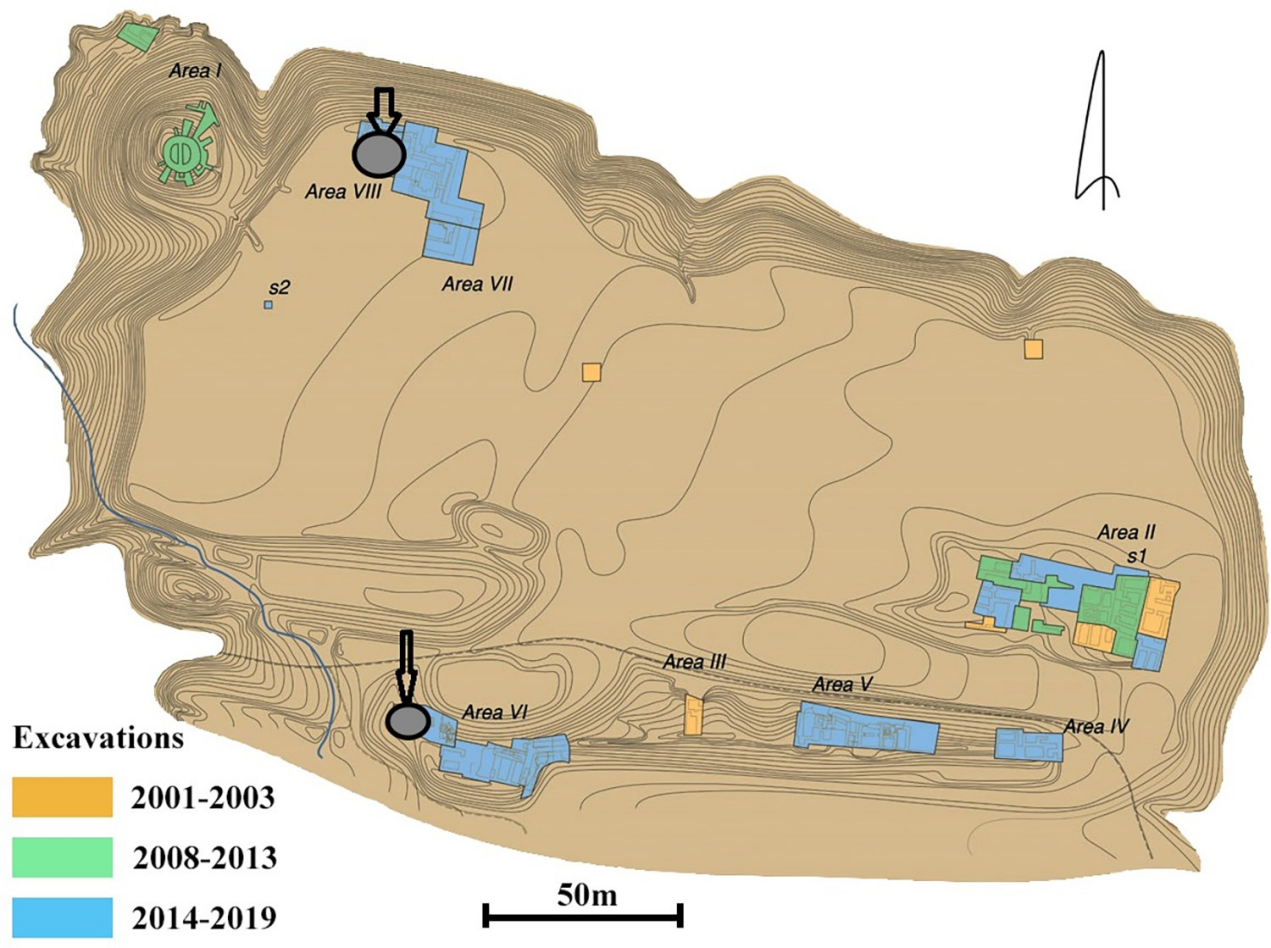
Sanjar-Shah. General plan with grey circles where samples were taken (adopted from [16] with Dr. Shenkar’s permission).

Sanjar-Shah (39.485359, 67.722012; 1063 m asl) is located around 12 km to the east of ancient Penjikent. The site was reported in 1947, but the first archaeological investigations were only conducted in 2001 by a German-Tajik archaeological team. Since 2014, excavations have been directed by Michael Shenkar and Sharof Kurbanov [14], with the support of The Society for the Exploration of EurAsia (Fig 3). In the eighth century, Sanjar-Shah was the second-largest settlement in the district [15]. Shenkar and Kurbanov [15] claim that the settlement was a significant place for enforcing political authority over Penjikent in this period; furthermore, scholars suggest that both cities were originally part of the same civic community. Sanjar-Shah, like Penjikent, consists of two main parts: the round tower and the main city (shahristan); the latter was originally divided into western and eastern. A large Sogdian palace was built in the western part in the 740s and lavishly decorated with wall-paintings and carved wood. Simultaneously a major urban development took place and areas along the southern city wall were built. The palace and probably some other areas were destroyed in the 770s-780s, but a second building phase took place shortly thereafter [16]. In contrast to Penjikent, mass finds of glazed ceramics and semi-circular sufa, typical of the ninth century, were recovered at Sanjar-Shah, suggesting a continued occupation in the early Samanid period.

#### Kuk-Tosh (10 – 12^th^ centuries AD)

Kuk-Tosh (39.492049, 67.639968; 1010 m asl) is located in the eastern part of the modern city of Penjikent and was discovered in 2015 at which time it was recognized as a pre-Mongol settlement. Through several years of continuous excavation, Aminov [17,18] revealed two occupation layers dated between the ninth and thirteenth centuries AD. According to recent studies, the settlement of ancient Penjikent was relocated to where it is now [17,19,20], as a newly established settlement after the Islamic conquest. Although, there are some finds of earlier pottery types and coins. Furthermore, as Aminov has shown, the inhabitants took an active part in trans-regional exchange. Dendroclimatic research has suggested a prolonged drought phase during the tenth century, which has been presented as one possible reason for migration slightly closer to the river [12].

#### Afrasiab (10 – 12^th^ centuries AD)

**Fig 4:**
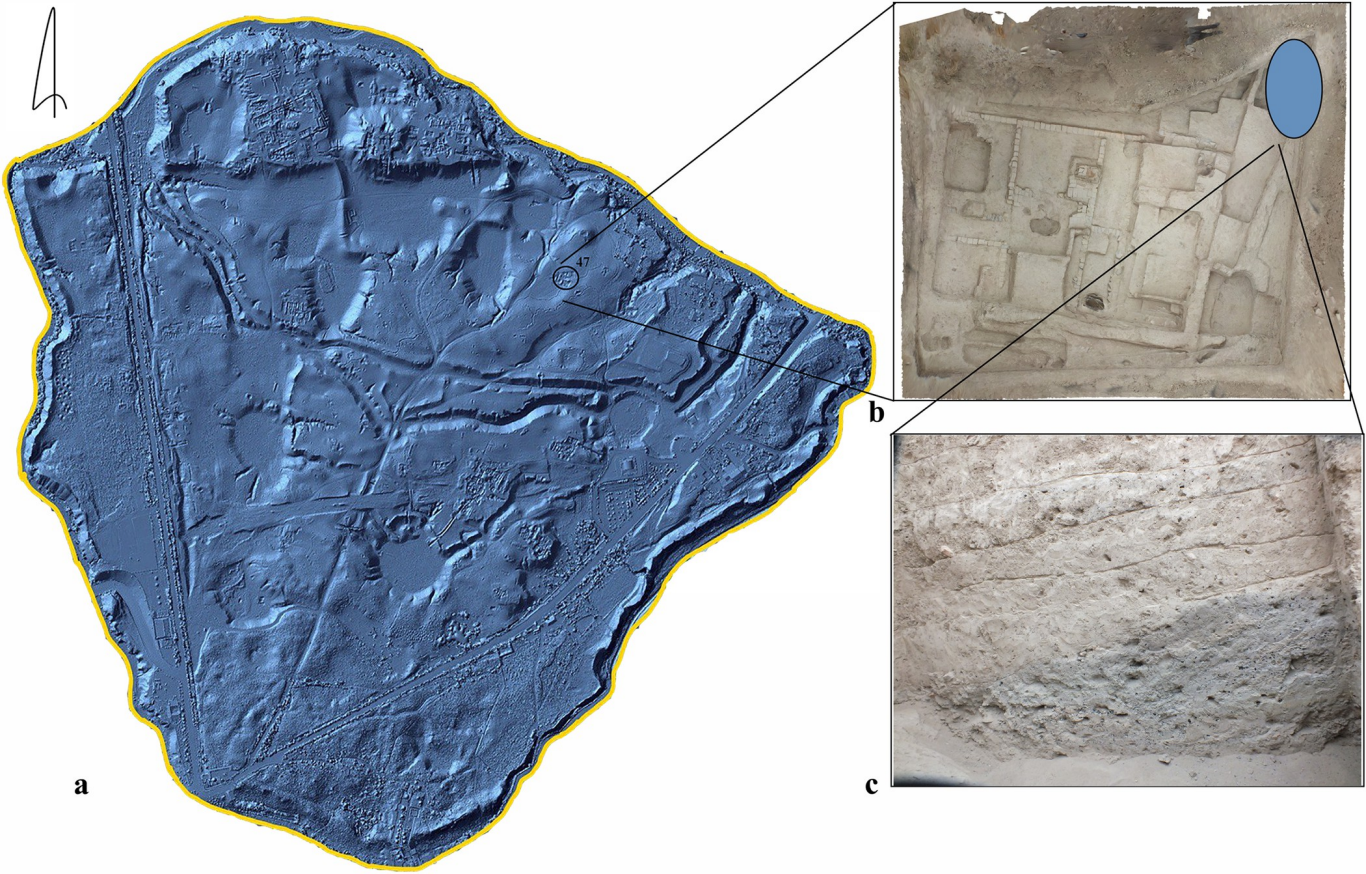
Afrasiab. a–Topographic map (map was produced within the Capacity Building of Cultural Heritage Tourism Resources Development Project of Samarkand, Uzbekistan that was planned as a part of Korea and Uzbekistan ODA (Official Development Assistance) Project from 2022 to 2026. The Samarkand archaeology institute, Korea cultural heritage foundation); b–Unit 47, c–cross section of a trash pit.

Afrasiab (39.67022, 66.987747; 712 m asl) was erected on a hilly terrain [21] located just north of modern Samarkand in Uzbekistan. The area of Afrasiab, at its peak, was almost 220 hectares [22,23] (Fig 4). Shishkina [8] claimed to have identified settlements near the citadel dating to the first quarter of the second millennium BC, based on burial traditions and fragments of handmade ceramic sherds. While archaeological survey shows that the settlement started expanding during the fourth century BC and its territory increased significantly during the third-first centuries BC, reaching a peak area of occupation in the sixth-seventh centuries AD [24]. At that time, only the northern half of Afrasiab was inhabited. However, it was expanded during the early Islamic and Samanid periods. The city itself and its suburbs, but not the citadel, were actually occupied at that time.

Historical sources state that the capital of the Samanid Empire (AD 875–999) was seated in Bukhara [23], but Afrasiab continued to be an important city in Transoxania [8,25]. Due to a lack of data, earlier scholars, e.g. Vyatkin [21] and Barthold [26], considered the Qarakhanid period a phase of decline for Afrasiab. The Qarakhanid period (AD 999–1220) seems more likely to have been a time of development, when Afrasiab became the center of production and trade [27]. Two separate Qarakhanid polities (Western and Eastern kingdoms or Qaghanates) emerged in AD 1040, the single political unit prior was initially centered in Kashgar [28]. Afrasiab transitioned from Qarakhanid to Khwarazmian rule in 1212, and its complete fall occurred in 1220, with the arrival of Genghis Khan [23]. Karev [23] wrote “the citadel of Samarkand was the place where the whole story of Qarakahnids over Transoxiana came to an end” (p. 145). In fact, the whole Afrasiab plateau was abandoned as the Mongol army interrupted the Djui-Arziz “Lead canal” [29]. The revival of an urban center at modern Samarkand, was undertaken only in the fourteenth century AD under the Timurid dynasty to the south of abandoned Afrasiab. In the framework of this study, we only studied materials dating to the tenth-twelfth centuries AD.

## Methodology

### Archaeobotany

During the Penjikent Archaeological Expedition in 2021 and 2022, a total of 38 sediment samples were collected, from Kainar (9 samples; 116.5 l), Penjikent (17 samples from three excavation areas: VI North, XI East and XXX; 411.5 l), Sanjar-Shah (9 samples; 176 l), and Kuk-Tosh (6 samples; 79 l). Sediment samples ranged from 4.5 to 61.5 liters in volume; in total, 783 l of sediment were floated from Tajikistan. Flotation work was also carried out at Afrasiab in 2019. We took only one sample (255 l) from a midden fill from a domestic context, approximately 500 m from the citadel area and roughly three meters below the surface. It was not possible to get more samples from Afrasiab as there were no ongoing excavations in the ancient city at that time. All analyzed sediments came from contexts that were already being excavated and would have been discarded without our collaboration.

We conducted water flotation on all sediment samples using an overflow tank system at the archaeological basecamp at Penjikent for samples from Tajikistan, while the Afrasiab sample was floated in Bukhara. Heavy fractions of each sample were collected down to 1.4 mm and light fractions down to 0.355 mm. The heavy fraction portions were sorted on site; while all light fractions were dried and transported to the Palaeoethnobotany Laboratory at the Max Planck Institute of Geoanthropology. In the laboratory, light fraction portions were sieved through meshes of 2.00, 1.40, 1.00, and 0.50 mm. Material smaller than 0.5 mm was not analyzed. After sieving, samples were systematically sorted under low magnification and diagnostic specimens were identified using atlases and seed identification manuals [e.g. 30–32].

We grouped fragmented seeds into several categories, including Cerealia, Legume, Millet, and unidentifiable seed fragments–none of which are counted in the totals. The Cerealia category includes all domesticated cereal grain fragments that were too small to identify as either wheat or barley; the Legume group includes fragments of domesticated Fabaceae that could not be distinguished between different genera.

### Radiocarbon dating

Two samples of specimens from Kainar and two samples of plant remains from Penjikent were AMS dated at the Woods Hole Oceanographic Institute Radiocarbon Laboratory, two samples from Afrasiab were run at the SUERC Radiocarbon Dating Laboratory, and two samples from Sanjar-Shah were run in FTMC Vilnius Radiocarbon Laboratory. All results were calibrated using OxCal v4.4.2 software [33,34] and the IntCal 20 curve [35].

## Results

### Radiocarbon and conventional archaeological dating

**Fig 5:**
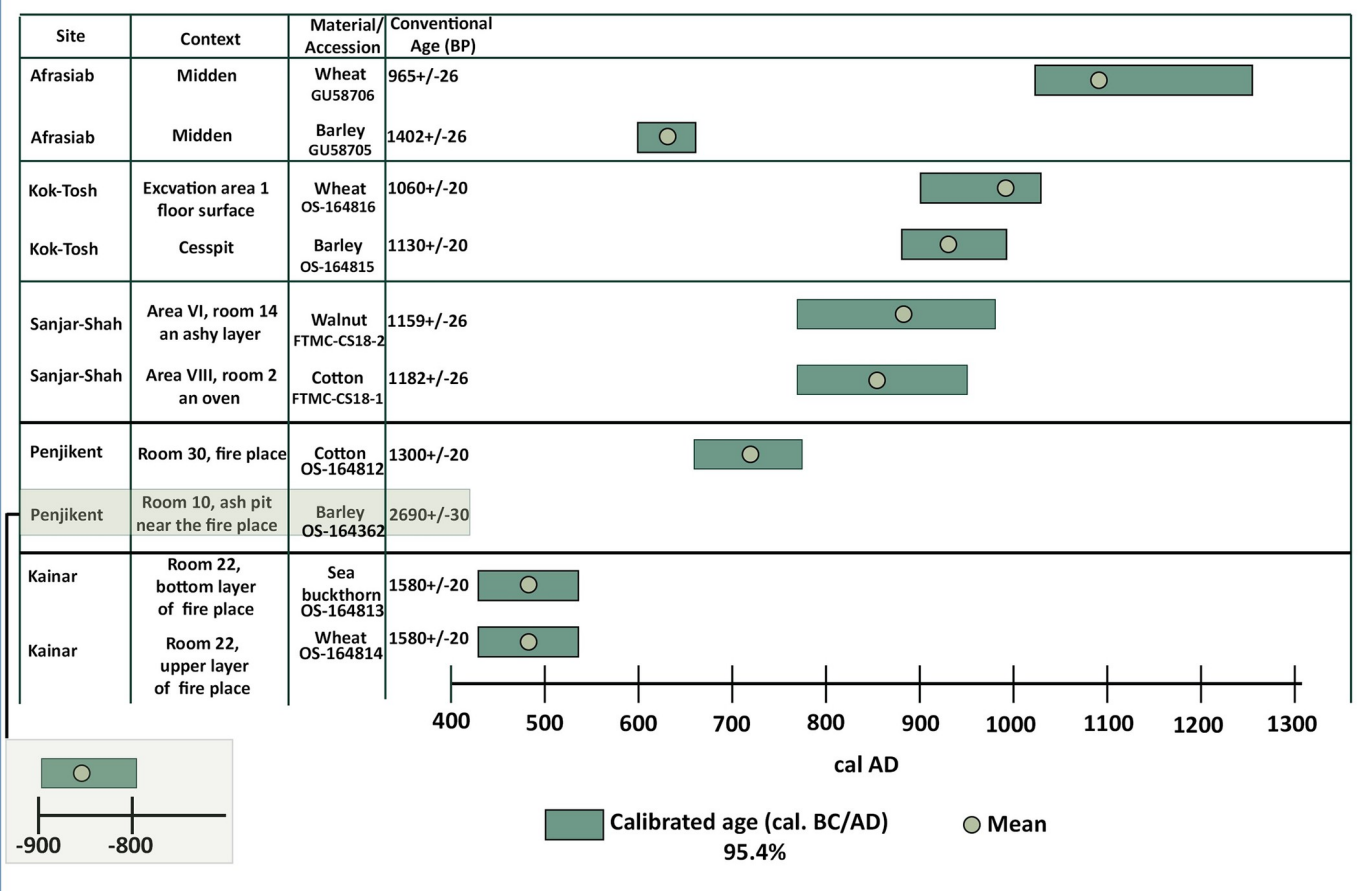
Radiocarbon dates of plant remains recovered from the five sites.

The results of radiocarbon dating seeds are shown in Fig 5. Based on 2-sigma calibration (95.4% probability), the dates from the Kainar campaign, confirmed artefact seriations, placing occupation at around the fifth-sixth centuries AD. Both samples came from the bottom and upper layers of a fire place in Room 22: a wheat (*Triticum aestivum*, OS-164813) grain and a sea buckthorn (*Hippophae rhamnoides*, OS-164814) seed were dated to between cal AD 429–546, with a mean of cal AD 486. A barley grain (*Hordeum vulgare*, OS-164362) recovered from Object VI-C, an ash pit in Room 10 at Penjikent, yielded a date of 2690 +/-30, calibrated to 902–803 cal BC. This date contradicts conventional archaeological dating of that context based on material culture, which places it at the beginning of the eighth century AD (before AD 722) (Table 2). While, a cotton (*Gossypium* sp., OS-164812) seed recovered from Object XI-B, a middle class house [for more details see: 36], in a fireplace in room 30, dated between cal AD 662–775, with a mean of cal AD 721 matched archaeological material dating.

Samples from Sanjar-Shah fall within the second half of the eighth and tenth centuries AD. A cotton seed (FTMC-CS18-1) recovered from the oven next to the western wall in Room 2, Area VIII, provided a date of between cal AD 772–950, with a mean of cal AD 847. The date on a walnut shell fragment (*Juglans regia*, FTMC-CS18-2) from the ashy filling next to the oven in Room 14, Area VI, ranged between cal AD 772–978, with a mean value of cal AD 883.

Almost all radiocarbon dates from Kuk-Tosh correlate with the conventional archaeological dating chronology compiled for the site based on ceramic and numismatic seriation. A barley grain (OS-164815) recovered from a cesspit at Kuk-Tosh corresponds to a calibrated range of cal AD 882–991, with a mean value of cal AD 930. While a wheat grain (OS-164816) from the floor surface of unit 1 ranged between cal AD 899–1026.

The Afrasiab midden, located in unit 47, dates to the tenth-eleventh centuries AD, based on ceramic seriation. However, radiocarbon data provided dates spanning half a millennium, possibly due to reuse of the midden through time. A barley grain (GU58705) from Afrasiab dated between cal AD 603–663, with a mean of cal AD 635; while a wheat grain (GU58706) correlated with dates of archaeological finds since it was dated to cal AD 1025–1258, with a mean value of cal AD 1094. These direct dates, in most cases, support the chronologies established on archaeological grounds (coins, pottery, and stratigraphy) (see Table 1).

**Table 1:** Conventional archaeological dating of archaeobotanical samples based on coins, ceramics, and building horizons.

Penjikent 2021 (Main Settlement)				
Sample #	Period	Unit	Context	Volume (Liters)
FSP4	First half of the 8th century AD	Object XXX	Room 21, burned layer	9.5
FSP5	AD 722	Object XI-B	Room 28, burned layer above broken bricks	35
FSP6	Before AD 722	Object VI-C	Room 10, fire place	4.5
FSP7	Before AD 722	Object VI-C	Room 10, ash pit next to the fire place	61.5
FSP8	Before AD 722	Object XI-B	Room 27, burned layer	42.5
FSP9	Before AD 722	Object XI-B	Room 27, burned layer below FSP8 (around -50 cm away from FSP8)	26
FSP11	Before AD 722	Object VI-C	Room 9 (Pandus—stairs) burned layer	4.5
FSP12	Before AD 722	Object XI-B	Room 30, Fire place	27
FSP18	mid-8th century AD	Object XXX	Room19, hearth	6
FSP19	8th century AD	Object XXX	Street, near the Room 19, ash filling	27.5
FSP20	8th century AD	Object VI-C	Room 10, above the second-floor level	20.5
**Total**				**264.5**
**Penjikent 2021 (Citadel–Kainar)**				
**Sample #**	**Period**	**Unit**	**Context**	**Volume (Liters)**
FSP1	6th century AD	Citadel	Room 19—Ash fillings next to the tandyr	36
FSP2	6th century AD	Citadel	Room 19—Area near the tandyr (burned soil)	6
FSP3	6th century AD	Citadel	Room 19 -sample from the profile wall (ashy layer)	6
FSP10	After AD 620	Under the northern wall of the citadel	Room 19 (it could a street), burned layer	12
FSP13	5-6th centuries AD	Under the northern wall of the citadel	Room 22. Fire place (upper layer - 1st)	10.5
FSP15	5-6th centuries AD	Citadel	Room 22. Fire place (bottom - 3rd)	16.5
FSP16	before 8th centuries AD	Citadel	Room 24.	13.5
FSP17	before 8th centuries AD	Citadel	Room 22.	10
**Total**				**110.5**
**Penjikent 2022 (Main Settlement)**				
**Sample #**	**Period**	**Unit**	**Context**	**Volume (Liters)**
FSP1	AD 750–770	Object XXX	Room 22, an oven	50
FSP2	Before AD 722	Object VI-C	Room 11, fillings from the second floor	92
FSP3	AD 750–770	Object XXX	Room 22, floor layer	46
FSP4	End of 7th century—before AD 722	Object XI-B	Room 31	3.5
FSP5	Before AD 722	Object VI-C	Room 12, floor	12.5
FSP6	End of 7th century—before AD 722	Object XI-B	Room 31, fillings from the khum (insitu)	17
**Total**				**221**
**Kuk-Tosh (2021)**				
**Sample #**	**Period**	**Unit**	**Context**	**Volume (Liters)**
FSK1	mid of 10th century AD	Cesspit	Pit 6	4.5
FSK2	mid of 10th century AD	Cesspit	Pit 6	11
FSK3	mid of 10th century AD	Cesspit	Pit 6	14.5
FSK4	mid of 10th century AD	Cesspit	Pit6	18.5
FSK5	mid of 10th century AD	Cesspit	Fire area	17.5
FSK6	10-12th centuries AD	Excavation area 1	Floor surface	13
**Total**				**79**
**Sanjar-Shah 2021**				
**Sample #**	**Period**	**Unit**	**Context**	**Volume (Liters)**
FSS1	9th century AD	Area VIII	Room 8, Tandyr filling (bottom)	31
FSS2	8th century AD	Area VIII	Room 8, Floor surface	11.5
FSS3	8th century AD	Area VIII	Room 8, Southern part of filling	6.5
FSS4	8th century AD	Area VIII	Room 8, North-western part of filling	5.5
FSS5	8th century AD	Area VIII	Room 8, Fillings of the lower layer	11
**Total**				**65.5**
**Sanjar-Shah 2022**				
**Sample #**	**Period**	**Unit**	**Context**	**Volume (Liters)**
FSS1	First half of the 8th century AD	Area VIII	Room 2, western wall, oven	16.5
FSS2	Second half of the 8th century AD	Area VI	Room 14, ashy layer next to the oven	85
FSS3	End of 7th century AD	Area VI	Room 12, under floor	9
FSS4	First half of the 8th century AD	Area VIII	Room 2, IBP (?)	0.5
**Total**				**111**
**Afrasiab 2019**				
**Sample #**	**Period**	**Unit**	**Context**	**Volume (Liters)**
FS1	10-12th centuries AD	Unit 47	Midden	255
**Total**				**255**

### Archaeobotany at Kainar

**Fig 6:**
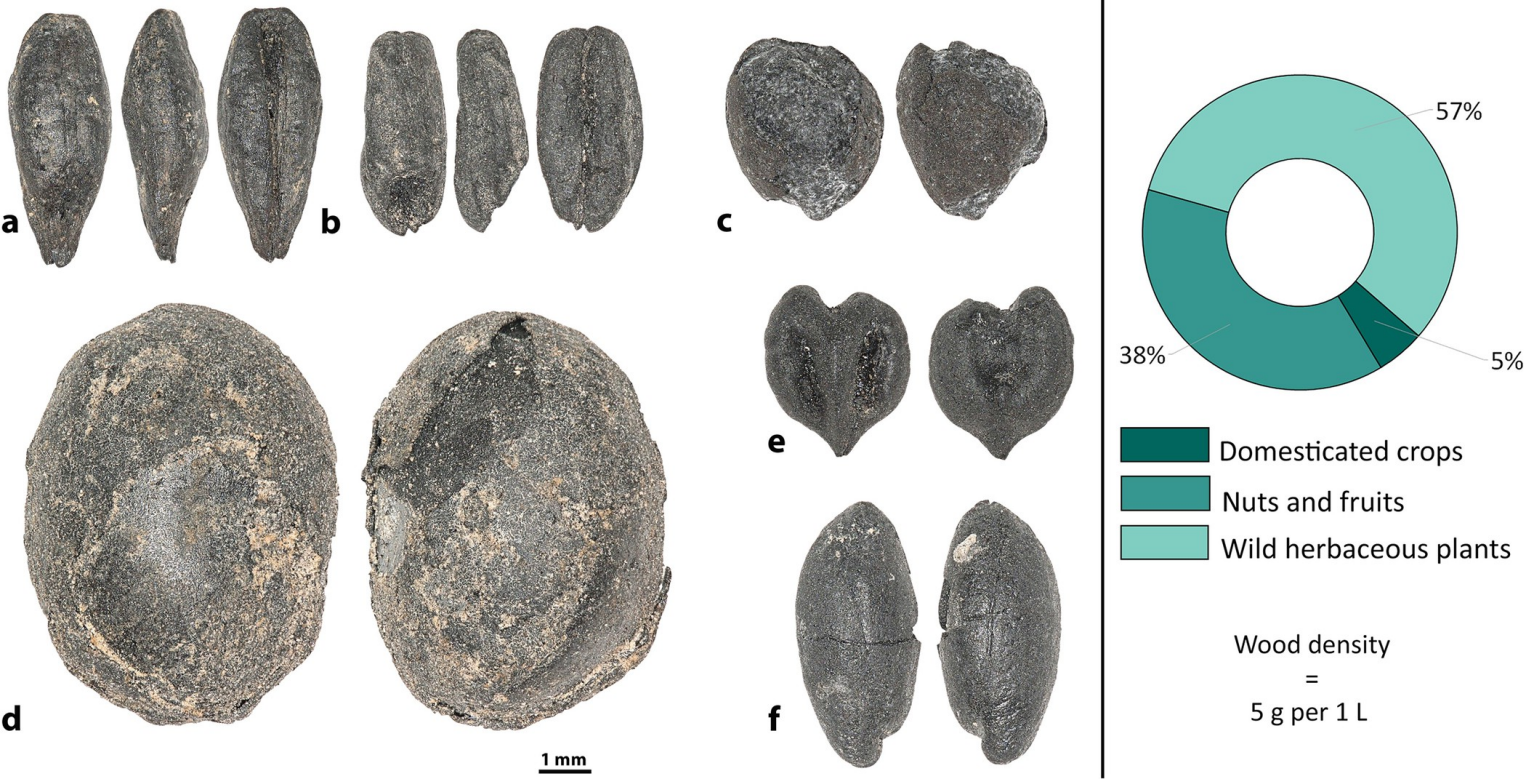
Plant remains recovered at Kainar. **a**–*Hordeum vulgare*, **b–***Triticum aestivum/turgidum*, **c–***Gossypium* sp., **d–***Vicia faba*, **e**–*Vitis vinifera*, and **f**–*Hippophae rhamnoides*.

The total density (seeds/liter of sediment) of material from Kainar was 2.04, and 580g of wood were recovered (S1 Table). Domesticated crops consisted of hulled barley (n = 4), free-threshing wheat (n = 6), a lentil (*Lens culinaris*, n = 1), a fava bean (*Vicia faba*, n = 1), and a cotton seed (*Gossypium* sp., n = 1) (Fig 6). In addition to annual crops, sea buckthorn (n = 83), Russian olive (*Elaeagnus angustifolia*, n = 1), grape (*Vitis vinifera*, n = 6), and apple/pear (*Malus/Pyrus*, n = 1) seeds were recovered, representing either cultivated or foraged wild fruits. Sea buckthorn seeds were relatively big compared to the wild form of the seeds in our reference collection, but seeds and fruit within this species span a wide range of sizes. Wild herbaceous plants represent more than 50% of the assemblage.

### Archaeobotany at Penjikent

**Fig 7:**
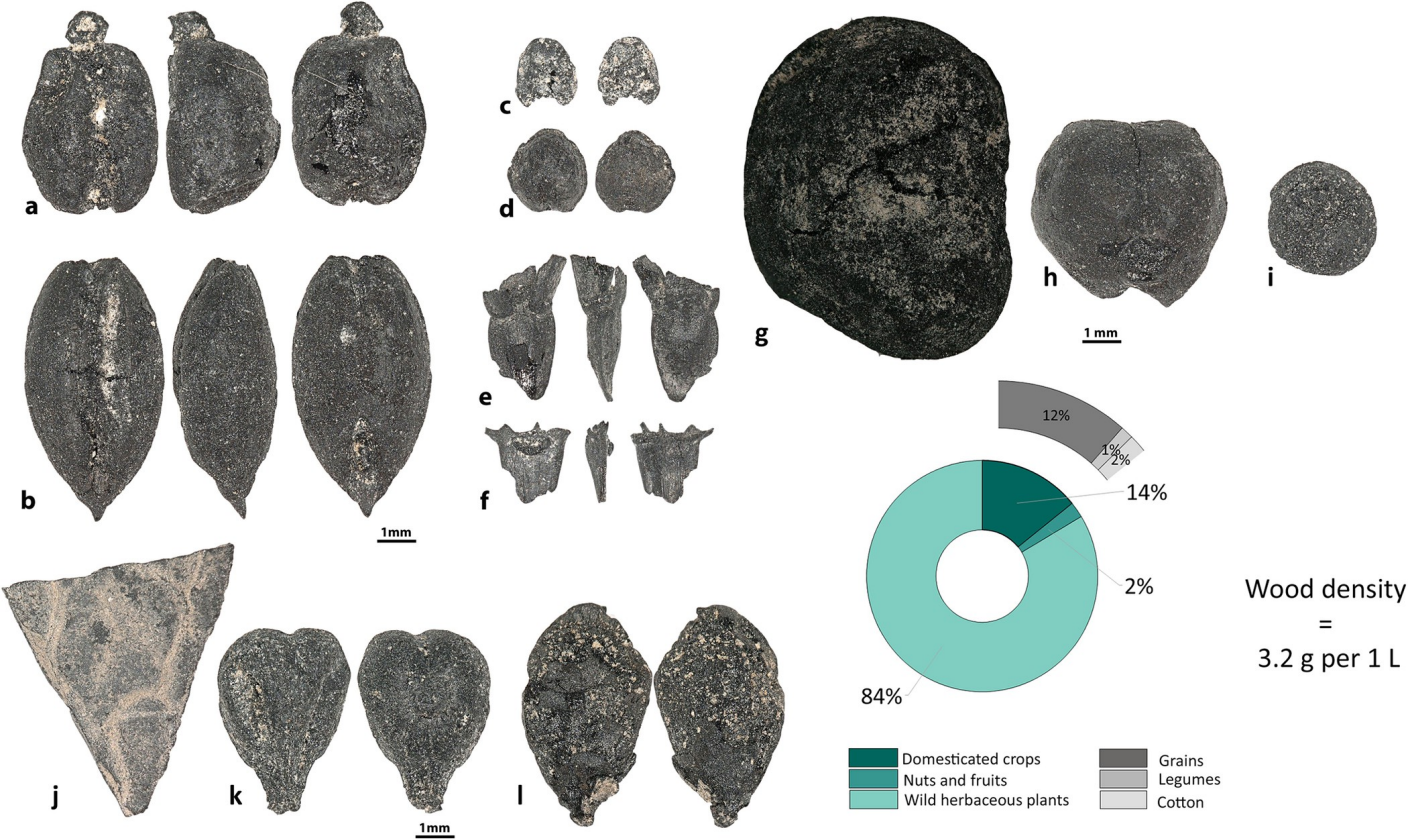
Plant remains recovered at Penjikent. **a–***Triticum aestivum*, **b**–*Hordeum vulgare*, **c**–*Setaria italica*, **d**–*Panicum miliaceum*, **e**–*Triticum aestivum* rachis, **f**–*Hordeum vulgare* rachis, **g**–*Vicia faba*, **h**–*Cicer arietinum*, **i–***Lens culinaris*, **j**—*Juglans regia*, **k–***Vitis vinifera*, and **l**–*Malus/Pyrus*.

A total of 3,324 carbonized seeds were recovered from Penjikent (S1 Table). In addition, 1,324.35 g of charred wood and 351 identifiable non-seed plant remains, such as rachises and culm nodes, were recovered. Four domesticated grain types, barley (n = 129), free-threshing wheat (n = 215), foxtail millet (*Setaria italica*, n = 13), broomcorn millet (*Panicum miliaceum*, n = 23), and undifferentiated millet grains (n = 3) were recovered. Wheat was the dominant crop, which was identified in abundance in Object 6 of C unit–the upper-class quarter. Legumes are represented by chickpea (*Cicer arietinum*, n = 6), lentil (n = 18), pea (*Pisum sativum*, n = 2), and fava bean (n = 10). In addition, 53 cotton seeds were identified (Fig 7). Cotton seeds have been recovered predominantly from the quarters of the low (Object XXX) and middle social classes (Object XI-B). Fruits and nuts comprised a small portion of the Penjikent assemblage, including grape (n = 19), apple/pear (n = 1), walnut shell fragments (n = 2), almond shell fragments (*Prunus dulcis*, n = 3), and melon (*Cucumis* sp., n = 2). Wild plants represent a large part (83.6%) of the assemblage.

### Archaeobotany at Sanjar-Shah

**Fig 8:**
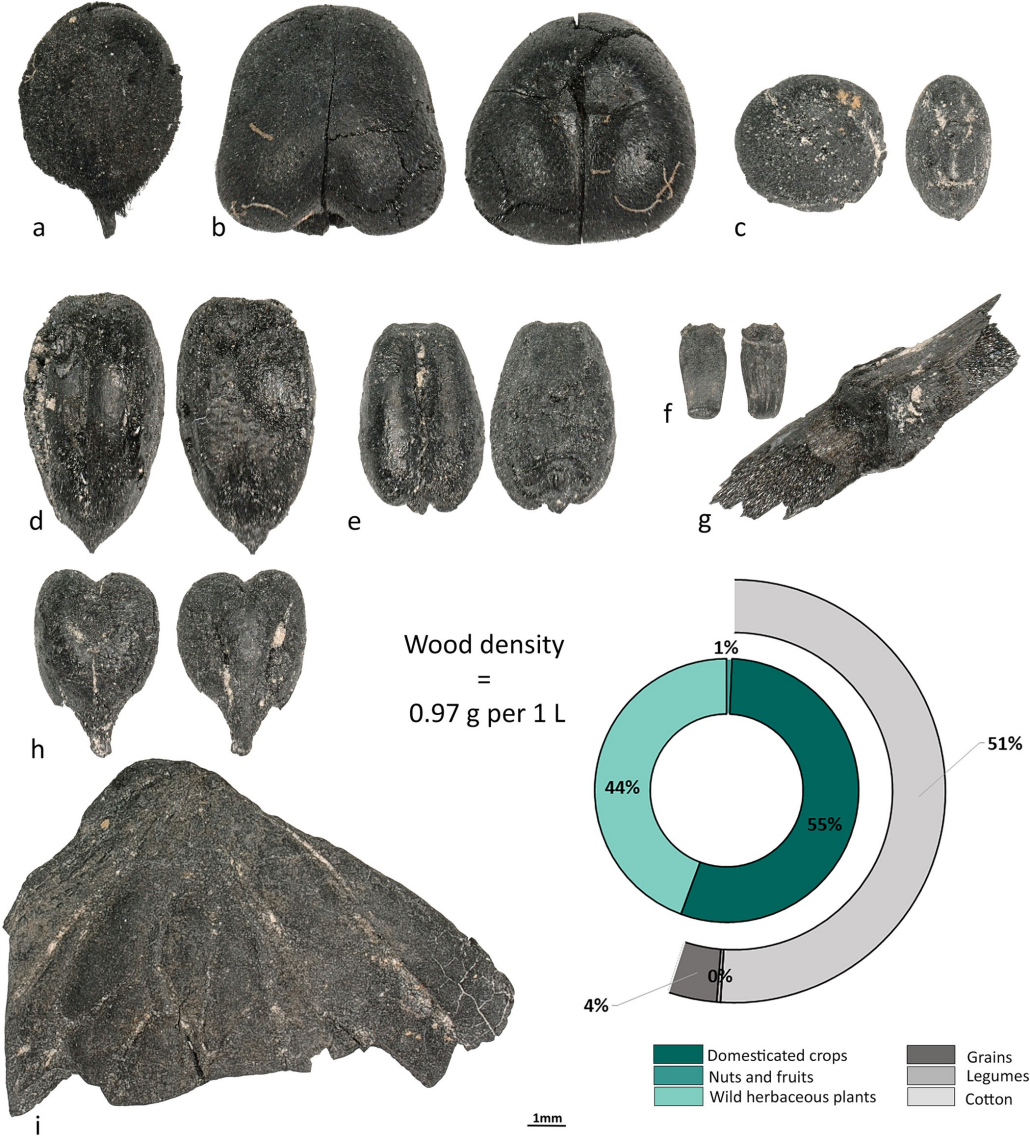
Plant remains recovered at Sanjar-Shah. **a**–*Gossypium* sp., **b**–*Vicia faba*, **c**–*Lens culinaris*, **d**–*Hordeum vulgare*, **e**–*Triticum aestivum*, **f**–*Triticum aestivum* rachis, **g**–culm node, **h**- *Vitis vinifera*, and **i—***Juglans regia*.

Of the 3,246 identified seeds from Sanjar-Shah, 55% were from domesticated crops, 0.64% from wild/cultivated fruits and nuts, and 44.36% from wild herbaceous taxa (Fig 8, S2 Table). The total density was 18.4 seeds per liter. Macrobotanical remains included barley (n = 68), wheat (n = 22), foxtail millet (n = 15), broomcorn millet (n = 2), undifferentiated millet grains (n = 4), pea (n = 3), lentil (n = 2), grass pea (*Lathyrus sativus*, n = 5), fava bean (n = 5), cotton (n = 1,662), grape (n = 4), walnut shell fragments (n = 14), and sea buckthorn (n = 1). Almost 99% of the cotton seeds were recovered from two samples from the oven next to the western wall in Area VIII—Room 2; while, 68% of the wheat grains were recovered from the ashy layer next to the oven in Area VI—Room 14. We also recovered 1,439 wild seeds, representing more than 35 taxa. The most numerous types belonging to *Carex* spp., Amaranthaceae, and *Trifolium* sp., with the most ubiquitous being *Chenopodium* sp. and Poaceae—all likely represent dung burning.

### Archaeobotany at Kuk-Tosh

**Fig 9:**
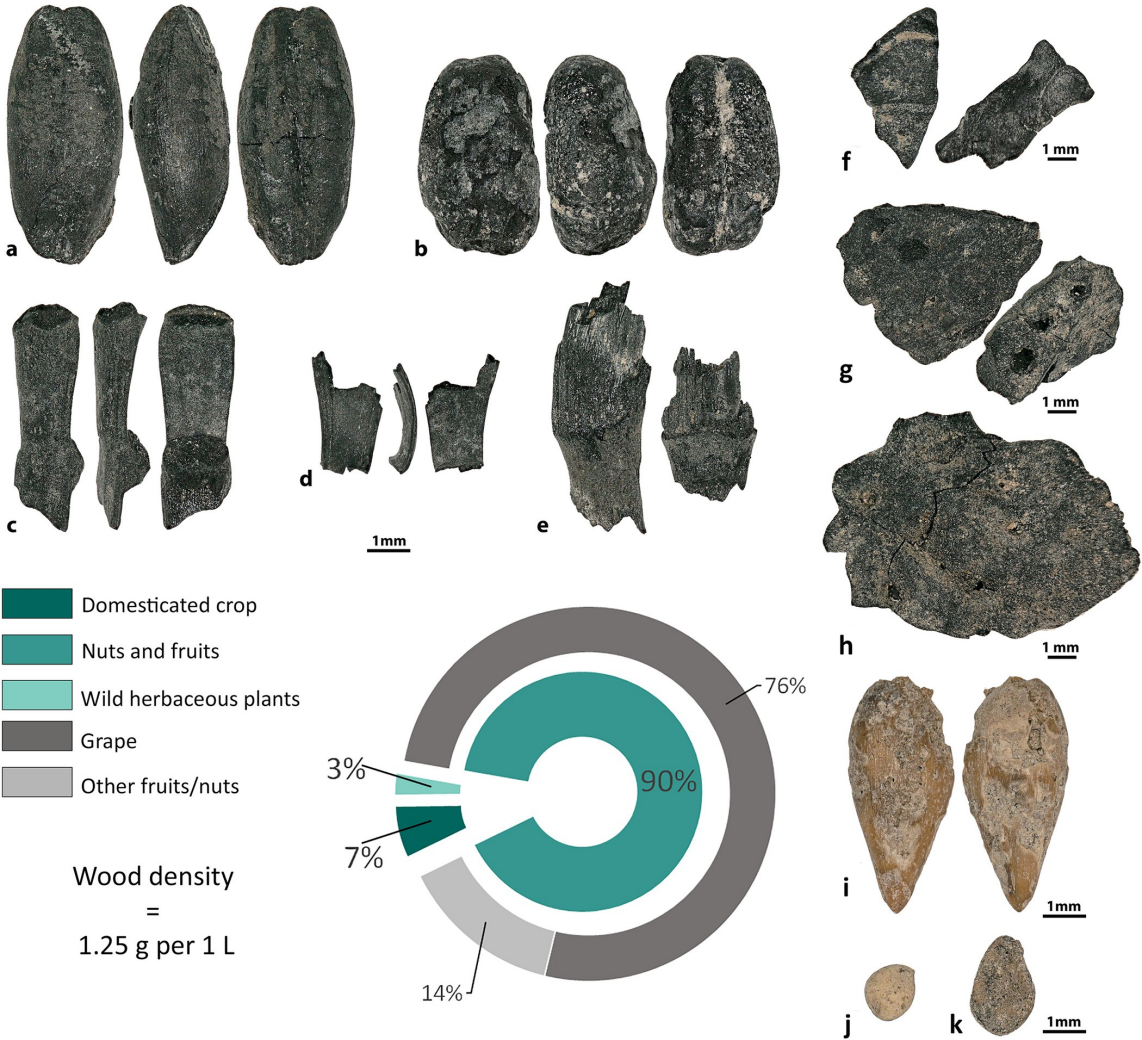
Plant remains recovered at Kuk-Tosh. **a**–*Hordeum vulgare*, **b**–*Triticum aestivum/turgidum*, **c**–*Triticum* cf. *turgidum* rachis, ***d****–Hordeum vulgare* rachis, **e**–cereal culm nodes, **f–***Juglans regia*, **g**–*Prunus dulcis*, **h**–*Prunus persica*, **i**–cf. *Punica granatum*, **j–***Ficus carica*, and **k**–*Morus* sp.

At Kuk-Tosh, floated sediment yielded 663 specimens (S3 Table), of which 90% were of cultivated and wild nuts and fruits, 7% were domesticated staple crops, and 3% were of wild herbaceous plants (Fig 9). While most seeds recovered from the cesspit were mineralized, a few carbonized grains and nutshell fragments were recovered from this context; most carbonized material came from the area around the fire and occupation floor of area 1. Most of the grape pips (99.6%) were preserved in a mineralized state; in addition, fig (*Ficus carica)*, mulberry (*Morus* sp.), and melon seeds were preserved and recovered mineralized. Carbonized nutshell fragments (such as walnut, peach (*Prunus persica*), and almond (*Prunus dulcis*), cereal crops, and their by-products were present in all samples at Kuk-Tosh. There were also three domesticated field crops–barley, wheat, and cotton. In total, 98.9 g of charred wood fragments (>2.0mm) were recovered from 6 samples.

### Archaeobotany at Afrasiab

**Fig 10:**
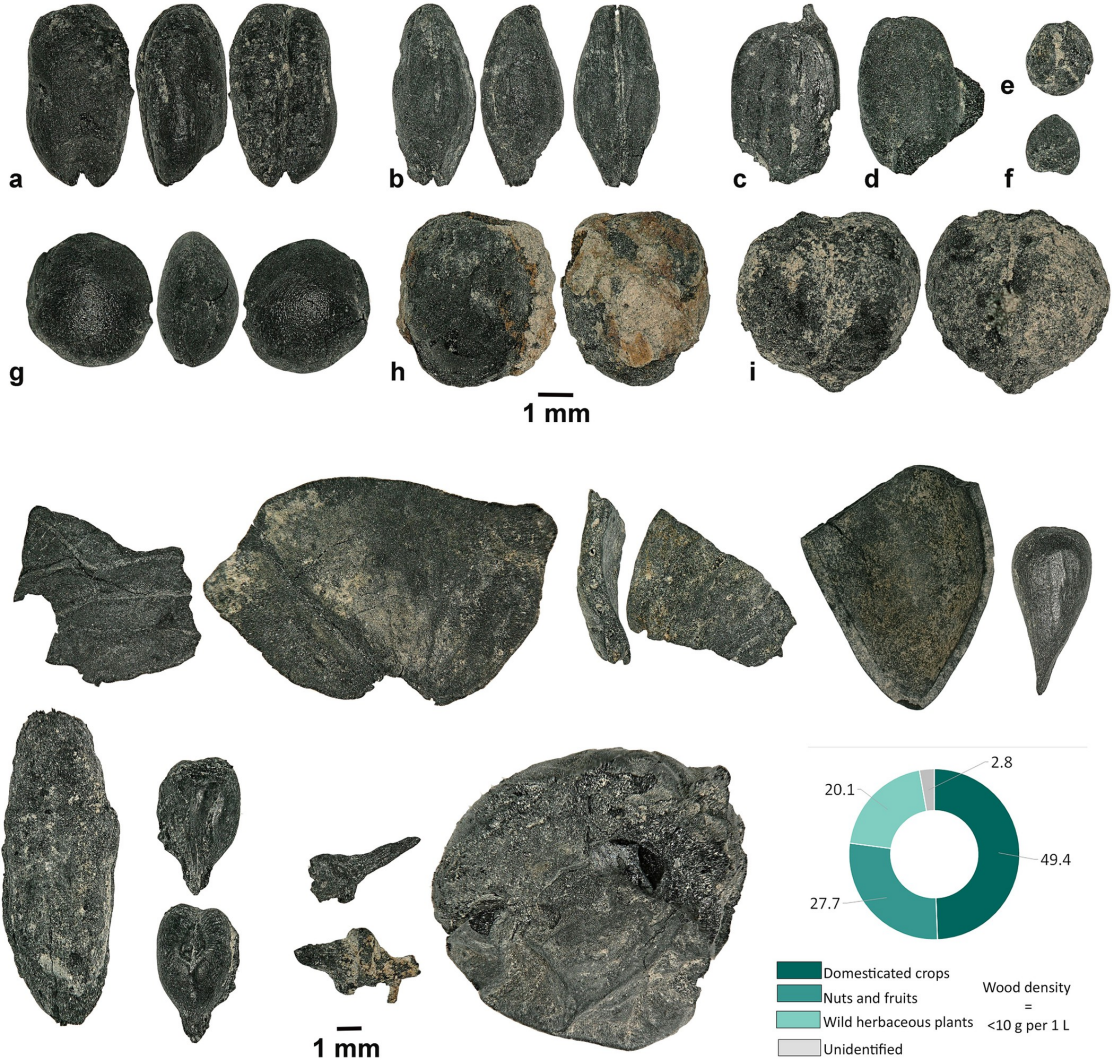
Plant remains recovered at Afrasiab. **a**–*Hordeum vulgare* var. *vulgare*, **b**–*Triticum aestivum*, **c, d**–*Oryza sativa*, **e, f**–*Panicum miliaceum*, **g**–*Lens culinaris*, **h**–*Pisum sativum*, **i**–*Cicer arientinum*, **j–***Juglans regia*, **k**–*Prunus armeniaca*, **l**–*Prunus dulcis*, **m**–*Pistacia vera*, **n**–*Malus domestica*, **o**–*Elaeagnus augustifolia*, **p–***Vitis vinifera* (pip), **q**–*Vitis vinifera* (pedicel), and **r**–*Vitis vinifera* (berry).

A total of 609 carbonized seeds and fruit parts were retrieved, as well as 18 mineralized seeds and 37 seed fragments (S4 Table). Domesticated field crops from Afrasiab account for 20.2% of the archaeobotanical assemblage. These include hulled barley (n = 21), free-threshing hexaploid and tetraploid wheat (*Triticum aestivum/turgidum*, n = 47), wheat (*Triticum* sp., n = 9), broomcorn millet (n = 2), rice (*Oryza sativa*, n = 3), lentil (n = 32), chickpea (n = 2), and pea (n = 4). Legume remains were present at a ratio of 1:3 to cereal grains; lentil is the most common pulse species recovered in the assemblage. Fruit and nut remains account for 29.2% of the whole assemblage (Fig 10). The most abundant fruit seed is apple/pear (n = 47), followed by grape pips (n = 23), full grapes (n = 6), and grape pedicles (n = 5). The nutshell fragments belong mainly to walnut (n = 51), apricot (n = 11), almond (n = 1), *Prunus* sp. (n = 10), Russian olive (n = 3), and pistachio (*Pistacia vera*; n = 2).

Seeds of wild herbaceous plants represent half of the archaeobotanical assemblage. Among these, 63% are *Thymelaea* sp. (n = 201) seeds; these are very small seeds with a smooth surface that makes them easy to move around in the soil and could, therefore, represent later intrusion. We speculate that these seeds were transported to the site in seed rain or by burning brush, as opposed to dung burning, as the density of wild plants is low (0.8 seeds/per liter). Moreover, many of the key species in this region usually associated with dung burning were not retrieved from the Afrasiab assemblage. Finally, *Chenopodium/Atriplex* (n = 19), *Carex* sp. (n = 1), *Trifolium* sp. (n = 2), Poaceae (n = 2), Panicoid (n = 4), *Rumex* sp. (n = 1), *Polygonum* sp. (n = 1), *Galium* sp. (n = 2), and Solanaceae (n = 31) were also recovered in small numbers.

## Discussion

### Economic plants of the upper part of the Middle Zarafshan Basin

The earliest evidence of agriculture thus far recovered in ancient Sogdiana comes from Sarazm, located 15 km west of the early medieval settlement of Penjikent and 45 km west of Afrasiab [37,38]. Sarazm is a proto-urban center in the Middle Zarafshan, with archaeological remains dating from the fourth to the end of the third millennium BC, and is culturally tied to populations in the southern Kyzyl-Kum Desert [38]. The archaeobotanical results from Sarazm illustrate that people were cultivating free-threshing wheat and hulled and naked barley (*H*. *vulgare* var. *nudum*), and foraging wild Russian olive, hackberry (*Celtis* sp.), sea buckthorn berry, wild pistachio, cappers (*Capparis spinosa*), and cherry (*Prunus* sp.) [38].

Our study shows that the diet of people in the cities of the upper part of the Middle Zarafshan became more diverse during the first millennium AD, results that contradict to Hermes et al. [39], who claim that people in the cities/towns expressed a limited dietary range in comparison to rural populations. The analysis of five assemblages of plant remains from the upper part of the Middle Zarafshan illustrates the continued cultivation of wheat and barley, and rice first appears in the assemblages only at the end of first millennium AD. Prior to this study, the only data regarding legumes from sites in the upper part of the Middle Zarafshan included: 1) peas mentioned in one Sogdian document from Mugh Mount (B8, line 9–10) [40–42], and 2) archaeobotanical peas reported from the Urtakurgan site [43]. In our study, we show that lentils (Kainar, Penjikent, and Afrasiab), peas (Penjikent, Kuk-Tosh, and Afrasiab), chickpeas (Penjikent and Afrasiab), and fava beans (Kainar, Penjikent, and Sanjar-Shah) were present. Finding lentils and chickpeas in Penjikent and Afrasiab is noteworthy for the Middle Zarafshan Basin, since they had not been recovered prior and were not mentioned in the textual sources for this region prior. Fava bean is a rare legume in Central Asia, and the earliest evidence comes from two sites in Turkmenistan–Togolok [44] and Adji-Kui [45]–dated to the late third and early second millennia BC. Fava bean has also been reported at Mugh, and the authors of the report suggested that the beans were morphologically similar to ones from Afghanistan [46]. They further suggested that the beans could have diffused to Tajikistan from Afghanistan and later spread across southern Central Asia. However, as can be observed in Figs 6–8, their shape and size vary considerably. Archaeobotanists working in the Levant have used geometric morphometrics accompanied by stable isotopes to argue that the size variation in ancient fava beans results from water availability during growth [47]. Although the fava beans in our study were likely an elite food and possibly not locally grown.

**Fig 11:**
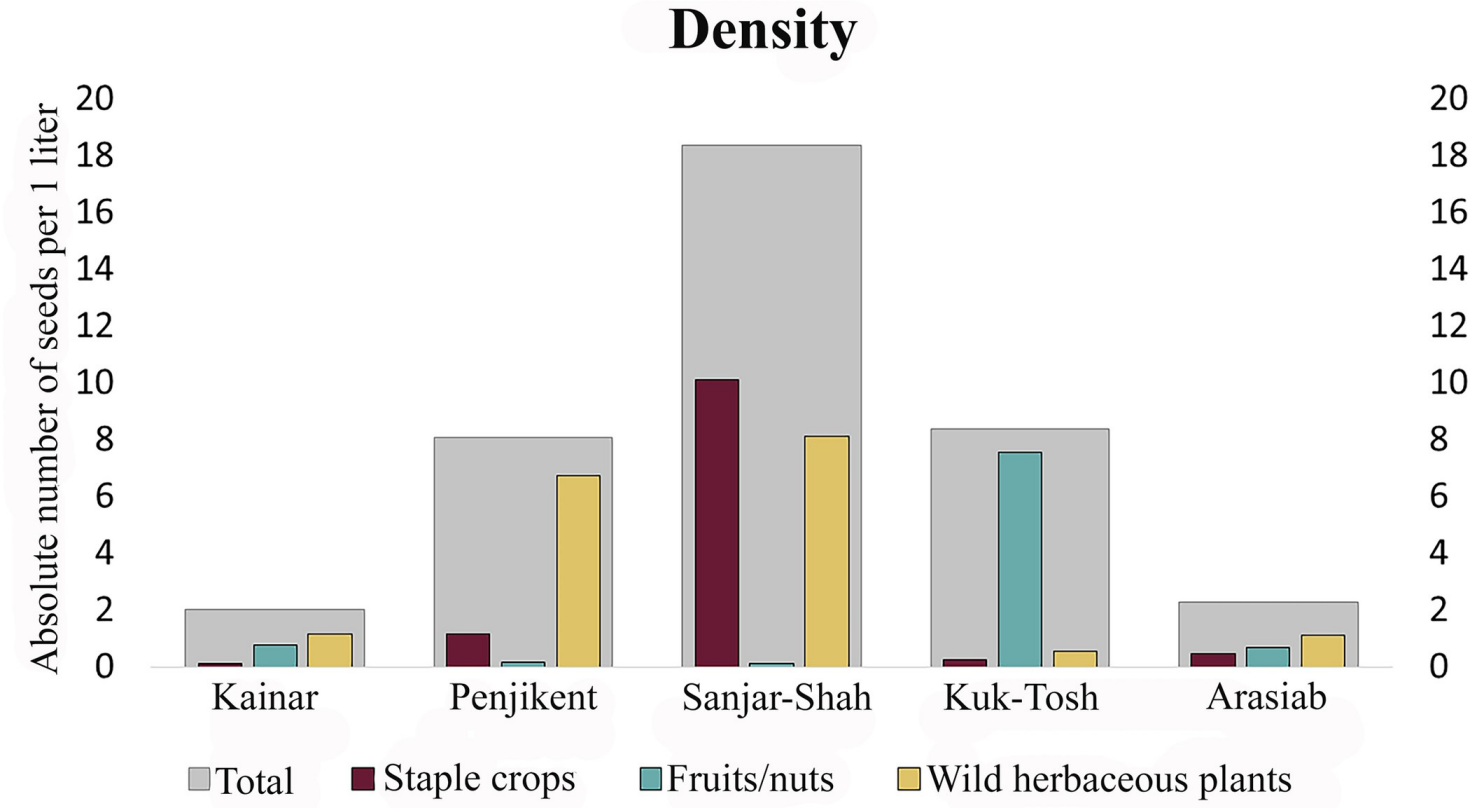
Density of three main categories of plants from the five assemblages.

**Fig 12:**
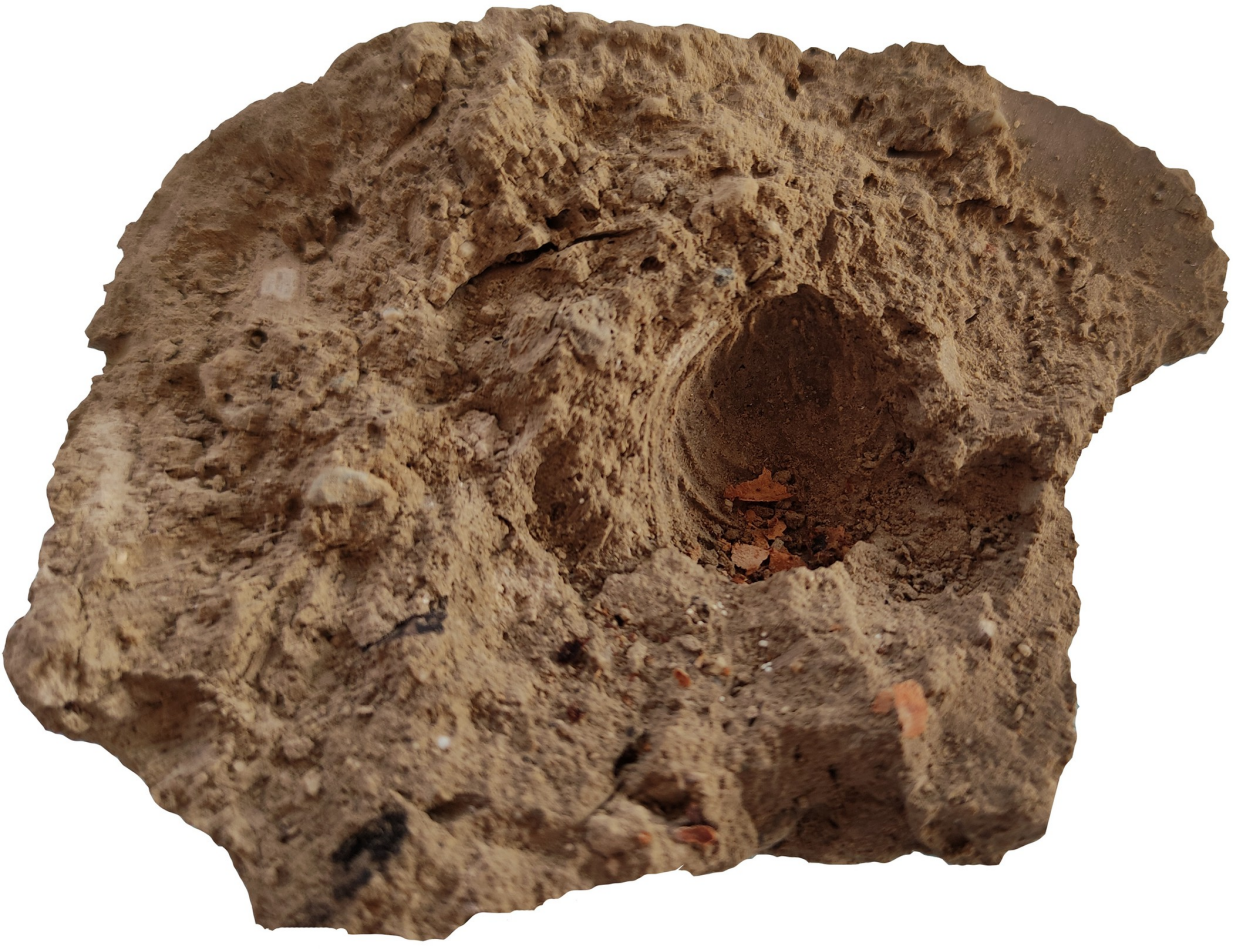
Peach stone imprint on the mudbrick from the XI-B object.

Spengler [48] and Schafer [49] each dedicated almost half of their books to showing the significance of fruits in the Central Asian economy during the first millennium AD. Although, the absolute counts (S1–S3 Tables) and densities (Fig 11) of fruit and nut remains in our study were low (except for Afrasiab and Kok-Tosh), this is presumably due to the fact that these remains are less likely to enter the fire and be preserved (e.g. Afrasiab) and due to the mineralized preservation mode (e.g. Kok-Tosh). On the basis of earlier published archaeological reports [e.g. 41,43,46,50,51], combined with the data that we present here, we suggest that grapes, apricots, peaches, and walnuts comprised the basis of arboriculture in the region. Danilevsky et al. [46] stated that they found peach and apricot stones at Mugh, concluding that the apricots were likely from a cultivated but small-fruiting form similar to those grown today in the Zarafshan valley and Bukhara oasis. Apricots and peaches were the most abundant hand-picked plant remains in the Hisorak settlement, located in the Upper Zarafshan region. Moreover, Danilevsky and his colleagues, analyzing full apple pome fragments, concluded that there were several apple varieties found at Mugh [46]. Recovered mural paintings in Sector XXIV, Room 1, show that peaches, melons, and grapes were the main attributes of feasting among the elites in Penjikent. We do not have enough evidence to conclude whether melons and peaches were also widely consumed among non-elites in the Penjikent intermountain corridor, but during excavation at Penjikent in 2021, we found a peach stone imprint on a piece of mudbrick from the XI-B object, a low-middle class quarter (Fig 12). The impression may indicate that only a small portion of the fruit seeds are actually entering the archaeobotanical record.

### Agriculture before and after the Islamic conquest in the upper part of Middle Zarafshan

Shenkar [4] states that infrastructural damage from the Islamic conquest at Sogdian cities was not as dramatic as it was during the Mongol invasion in the thirteen century AD, however, the damage to the agricultural hinterland must have been considerable. Shenkar [4] writes, “the main reason for the disappearance of the Sogdian civilization was the dismantling of the Sogdian self-governing civic communities by the Arabs”. Gibb [52], pointing to the journey of Xuanzang in AD 630, stated that Sogdiana was divided into twenty-seven states under separate rulers. Most of the urban settlements (e.g. Paykend, Bukhara, and Afrasiab) were rebuilt and reoccupied after the Islamic invasion, Penjikent being partially an exception, the city was reinhabited after 738, lasting until 770. The extent of the Islamic influence on agriculture in Central Asia has long been a subject of interest to historians and archaeologists [53]. Watson [54,55] argued for an Islamic Green Revolution, envisioning an intensification and diversification of agriculture across southwest Asia and the eastern Mediterranean starting after the political conversions to Islam. Watson [55] stated that Arabic-speaking people introduced and/or intensified the cultivation of 18 crops across the Islamic World. As has been recently discussed [56], only a few crops from Watson’s list, notably cotton, rice, watermelon (*Citrulus lanatus*), tetraploid free-threshing wheat (*Triticum turgidum* spp. *durum*), spinach (*Spinacia oleracea)*, and eggplants (*Solanum melongena*), are ecologically suited for cultivation in Central Asia. Mir-Makhamad and Spengler [56] propose that some limited of agricultural diversification may have taken place starting in the fifth-sixth centuries AD in the territory of southern Central Asia. But they cushion this statement by noting that the consequences of the Islamic conquest on agriculture should be considered in the framework of specific regions and local ecological conditions. Archaeobotanical data show that cotton was produced before and after Islamic conquest; whereas, rice was mainly recovered from cultural deposits post-dating the Islamic conquest in Afrasiab, Bukhara, and Paykend. Based on the available data from the core areas in Central Asia, free-threshing tetraploid wheat was likely reintroduced into southern Central Asia in the second half of the first millennium AD. There is still no solid archaeobotanical evidence of watermelon cultivation from the Zarafshan region before the Islamic conquest. Watson (1983) mentioned eggplant cultivation in the region of Samarkand in the first millennium AD, but currently archaeobotanical studies have only recovered eggplant seeds at Bukhara [59]. Similarly, spinach remains have not yet been reported, but are extremely unlikely to be identified archaeobotanically.

Contrasting data from these five studied sites is complicated by differences in depositional contexts, which affect plant preservation. The material from the pre-Islamic (before AD 712) and transitional period (eighth century AD) contexts exclusively comes from burning and cooking, while, plant remains recovered from the cesspits in Kuk-Tosh and Afrasiab resulted from kitchen refuse and human excrement. Fruits are quite rare as carbonized findings, because they are consumed raw and are not necessarily disposed of in fires. Nut shells were recovered in the Afrasiab midden and the Kuk-Tosh cesspit in a carbonized state. It is unlikely that we have identified the earliest introduction of mulberry, fig, and pomegranate, as art historical evidence [15] shows that Sogdians were at least familiar with pomegranate (for a summary of early depictions of pomegranate in Central Asia, see [57]). Figs, mulberries, and pomegranates were either imported and associated with an elite segment of society prior to the Islamic conquest, or preservation biases make the fruit archaeobotanically invisible prior to the recovery of mineralized remains from ninth century AD cesspits.

While further archaeobotanical work is needed, we propose that there were two waves of crop diversification in the first millennium AD of the Middle and Lower Zarafshan. The first wave appeared in the Middle Zarafshan Basin from the fifth to seventh centuries AD–during Sogdian control–marked by construction of irrigation systems [7], urbanization, and population increase. Large-scale collective labor projects, such as the Bukhara great wall and the construction of canals across the Zarafshan started in this period. The second wave was only in the ninth century AD–during the Samanid regime–when it is likely that fruits, such as mulberries, figs, pomegranates, and watermelons were brought under cultivation (recognizing the data biases noted in the previous paragraph), possibly also new herbs, spices, and dyes (Table 2).

**Table 2:** Economic plant remains from sites along the Zarafshan River.

	masl	Domesticated cereals						Domesticated legumes					Fiber/oil crops		Spices/ dyes					Nuts			Fruits/berries															
Site/Crop		Wheat	Barley	Broomcorn millet	Foxtail millet	Millet	Rice	Lentil	Mung bean	Pea	Chickpea	Fava bean	Cotton	Flax	Coriander	Sesame	Pepper	Cumin	Sumac	Pistachio	Almond	Walnut	Grape	Apple	Pear/Apple	Apricot	Peach	Plum	*Prunus* sp.	Fig	Mulberry	Sea buckthorn	Melon	Watermelon	Russian olive	Pomegranate	Barberry	Hackberry
Kainar (AD 429–546)	1022	x	x					x				x	x										x		x							x	x		x			
Penjikent (AD 662–775)	1051	x	x	x	x	X		x		x	x	x	x								x	x	x		x		x											
Sanjar-Shah (AD 700–900)	1063	x	x	x	x	X		x		x		x	x									x	x									x						x
Kuk-Tosh (AD 882–1026)	1010	x	x							x			x								x	x	x	x			x		x	x	x		x			x		
Mugh (AD 700–722)	1362	x	x	x								x	x										x	x		x	x											x
Urtakurgan (AD 700–800)	1516	x	x							x																												
Chilkhudzhra (AD 700–800)	1518	x	x			X														x		x				x	x	x									x	
Afrasiab (400–100 BC)	710		x	x																																		
Afrasiab (AD 600–1200)	710	x	x	x			x	x		x	x									x	x	x	x		x	x									x			
Bash-Tepa (171BC-AD 58)	201	x	x																				x															
Bukhara (AD 261–532)	225	x	x	x	x								x										x															
Bukhara (AD 663–775)	225	x	x	x					x																													
Bukhara (AD882-991)	225	x	x	x	x	X	x	x		x			x	x	x	x	x	x	x		x	x	x	x	x		x			x	x		x	x	x	x		
Paykend (citadel) (401–106 BC)	216	x	x	x	x			x																														
Payend (citadel) (AD 1–400)	216	x	x	x	x																																	
Paykend (Shahristan) (AD 897–1199)	218	x	x			X		x			x			x						x		x	x		x	x												
Paykend (Rabat) (AD 980–1223)	208	x	x	x	x	X	x	x		x			x										x		x				x				x		x			

### Agriculture on the Silk Road in the first millennium AD

Based on available data, we believe that agriculture fueled demographic and economic growth along the Zarafshan. Historical references to mercantile activities of the Sogdians can be found in the Sogdian Ancient Letters, the Gaoseng Zhuan (*Memoirs of Eminent Monks*) (AD 530), and *Christian Topography* (AD 547–550) to name just a few. These ancient texts suggest trade not only with northern Central Asia and China but also with India and southeast Asia [7]. Those merchants could have procured exotic products, like Indonesian pepper (likely *Piper nigrum*) or camphor (*Cinnamomum camphora*), both mentioned in the Sogdian Ancient Letters [58]. Nonetheless, the stereotype of the Sogdian merchant does not sum up the reality of their economy, which we suggest was based on agriculture. Our study shows that cereals and legumes were the main plant foods, supplemented by fruits that were likely grown in local Sogdian gardens.

The Zarafshan Oasis was one of the largest socio-economic centers within Central Asia, due to the well-developed irrigation system that supported agriculture, cattle-breeding, and craft production [59]. Comparing archaeobotanical data from the upper part of the Middle and Lower Zarafshan, cereals were recovered at all sites in both regions (Table 1). Although, legumes are less well represented in archaeological contexts than cereals, five legumes were recovered from sites along the river. Fava bean was recovered only in the upper part of Middle Zarafshan, and mung bean was recovered from Bukhara in the Lower Zarafshan. By the end of the twentieth century, almost 70% of irrigated land in Uzbekistan was planted with cotton, in Bukhara– 62%, and in the Kashka-Darya Oasis– 2,000 hectares [60]. Based on archaeobotanical data, cotton processing took place in both regions starting in the first half of the first millennium AD. However, there were no cotton fields (unlike Samarkand) in the Penjikent district in the Soviet era. Penjikent was considered to be ecologically better suited for fruits, tobacco (*Nicotiana tabacum*), and rice; although, it was possible to grow cotton there, it was considered less profitable under the Communist system.

## Conclusion

Archaobotanical studies conducted at five sites/areas located along the upper part of the Middle Zarafshan River document economic plant introductions in the first millennium AD. The main botanical components of the diet were cereals (free-threshing hexaploid and tetraploid wheats, barley, broomcorn and foxtail millets, and rice), legumes (lentil, pea, fava bean, and mung bean), fruits (peaches, apricot, melon, grape, pomegranate, Russian olive, fig, mulberry, and sea buckthorn), and nuts (walnut, almond, and pistachio). Crops continued to diversify in the Middle and Lower Zarafshan over time, likely tied to the intensification of irrigation systems, expansion of trade, establishment of reliable markets, urbanization, and population growth. We suggest that there may have been two independent waves of crop diversification, one in the fifth-seventh centuries in the Middle Zarafshan region and another in the ninth-tenth centuries in urban settlements along Middle and Lower Zarafshan, despite a dry climatic phase reported for the region.

Comparing archaeobotanical data from the two regions, we reveal that: 1) fava bean was likely cultivated in the upper part of the Middle Zarafshan; 2) cotton was introduced to the Zarafshan basin during the first half of the first millennium AD–before the Islamic conquest; and 3) walnut and grape remains were the most frequently recovered long-generation perennial crops in both regions. We also show that grape, walnut, peach, pomegranate, almond, and apricot were important perennial crops cultivated in the Zarafshan Basin in the first millennium AD. Despite the fact that fig, mulberry, and pomegranate remains were only recovered from the cultural deposits after the Islamic conquest, it is unlikely they represent the earliest introduction, since there is a high risk of preservation biases. More archaeobotanical studies in this region are needed, and a similar study to this one should be carried out in the Upper Zarafshan region, a high-elevation region, where sites are at risk because of the increasing rates glacial melt.

## Supporting information

S1 Table Kainar and Penjikent, Tajikistan (2021–2022). (XLSX)

S2 Table Sanjar-Shah, Tajikistan (2021–2022). (XLSX)

S3 Table Kuk-Tosh, Tajikistan (2021). (XLSX)

S4 Table Afrasiab, Uzbekistan (2019). (XLSX)
